# How Strong Is the Case for the RNA World?

**DOI:** 10.3390/life16101633

**Published:** 2026-09-30

**Authors:** Paul G. Higgs

**Affiliations:** Department of Physics and Astronomy, McMaster University, Hamilton, ON L8S 4K1, Canada; higgsp@mcmaster.ca

**Keywords:** RNA world, ribozymes, non-enzymatic replication, protocells, metabolism, homochirality

## Abstract

The RNA World theory was proposed as a way to explain the origin of the DNA-RNA-Protein relationship found all current cells. It has been greatly strengthened by experimental work on ribozymes and non-enzymatic replication, and has become the most widely accepted theory for the origin of life. But how much do we really know? Within the science community there remain arguments that something else is required before RNA, or that we should not focus on RNA as a single central player. From outside, there are claims that scientists are clueless about the origin of life and that biological information can only be explained by intelligent design. Here, I review recent work showing the strength and coherence of the case for the RNA World. Science can clearly defeat the argument for intelligent design. There is substantial progress with experiments demonstrating many of the requirements for an RNA-first Origin of Life, but important issues remain to be understood.

## 1. Introduction—The RNA World and Its Critics

The first proposals that RNA played a central role in the origin of life came in the early days of molecular biology when it became apparent that rRNA, tRNA and mRNA were all required for protein synthesis [1,2,3]. This idea became much firmer with the discovery of naturally occurring ribozymes [4,5], with the demonstration that the active site of the ribosome is made of RNA [6,7], and with the arrival of experiments on non-enzymatic RNA replication [8,9] and polymerase ribozymes [10,11]. Today the RNA-first theory is probably the most widely accepted theory for the Origin of Life [12,13,14,15], but there remain significant questions. Here, I discuss the steps that are required for an RNA-first OoL and ask to what extent these have been convincingly demonstrated.

Arguments for an RNA-first OoL focus on RNA as though it is the central or unique player in the story, but there are also many counter-arguments. Maybe there was something else before RNA, such as an alternative replicating polymer which was later replaced by RNA. Maybe a metabolism was established before RNA was synthesized. Maybe there were self-reproducing lipid micelles with compositional information. Maybe a process of chemical evolution was necessary before RNA arose. Maybe lipid vesicles were required. Maybe there were peptides alongside RNA. Maybe there were all sorts of other essential molecules. Despite all these additional possibilities, the essence of the RNA World idea is that RNAs acted both as genes and catalysts. If that is true, as I believe it is, it seems fair to think of RNA as the central player and to speak of an RNA World.

Table 1 summarizes the steps in the case for the RNA World. For each step I have tried to give a grade for the strength of the case. Very Strong means that current evidence is already clear beyond reasonable doubt. Strong means that we have good current evidence, but that significant questions remain and additional experimental demonstrations will be necessary. Weak means that we are still unclear about important questions. These grades are a personal assessment of the overall state of the field and I do not mean them as a criticism of the work of any particular research group.

From outside the OoL scientific community, there are claims that science is “clueless” to explain the OoL [16]. These claims have an underlying religious motivation. In particular, Meyer [17] has claimed that science cannot explain the existence of biological molecules with functional information. Functional molecules are claimed to be so rare that they could not arise by chance, and so they must have been created by an intelligent designer. He also claims that prebiotic evolution is impossible, and that evolution cannot explain the origin of functional molecules. I will show that these arguments for intelligent design are false. In fact, they argue against the steps in the case for the RNA World where the evidence is already Very Strong. Evolution is essential as a means to give rise to functional molecules. Therefore it is necessary to consider how evolution operated in the context of the earliest stages of life.

Unfortunately there is a large amount of ignorance about science in the general public and among political leaders. I therefore feel it is up to scientists to state the scientific case clearly. As evolution and OoL are a prime focus for anti-science arguments, the scienctific case in these areas is of high importance. So it is worth spending some time to lay out this case carefully. Here, I present the scientific case in the context of the RNA World because I believe this is the strongest scientific explanation that we have, and therefore the best way to counter anti-science arguments. But clearly it is not true that any scientist who rejects creationism and intelligent design must also be a supporter of the RNA World. There are many alternative scientific views in the OoL community. This article has the joint aims of addressing the claims made for intelligent design and also assessing the merits of the RNA World view in comparison to other scientific possibilities. I hope it is possible to achieve these two aims in a single article.

## 2. The Ribosome and the Central Dogma

The idea that RNA was important for the origin of life already seemed likely as early as the 1960s. Rich [1] and Orgel [2] pointed out that nucleic acids could act as templates for synthesis of a complementary strand, and hence could catalyze their own replication, whereas proteins could not. In discussing the translation process and the origin of the genetic code, Crick [3] was “struck by the considerable involvement of non-informational nucleic acid”. This was before the demonstration that RNA sequences could be catalysts and before it was known that the active sites in the ribosome were made of RNA. Current knowledge of these things makes it certain that RNA was very important very early in the history of life. As cells with ribosomes obviously did arise very early, we can frame the question of the OoL as “how did we get to the stage of cells with ribosomes?”

It may be useful to distinguish several different levels of claim about the role of RNA in OoL. I will call the weakest claim “RNA Before Proteins”. This simply says that RNA replication preceded synthesis of encoded proteins (although this does not exclude the existence of non-encoded peptides). The ubiquity of ribosomal protein synthesis in current cells makes it virtually certain that this claim is true. Therefore, I rate this aspect of the case as Very Strong.

A somewhat broader claim is “RNA World”, by which I mean that RNA sequences acted as both genes and catalysts at an early stage of life. In particular, I include here the claim that there were RNA polymerase ribozymes that copied RNA sequences. There could also have been ribozymes with other functions that were replicated by the polymerase ribozymes. The ribosome and the central dogma make a case for “RNA World” that is almost as strong as for “RNA Before Proteins”. The translation system depends on ribosomal RNAs and transfer RNAs of many types, and it is almost impossible to imagine how multiple quite long RNAs with specific sequences could arise and be replicated if there were no RNA polymerase ribozymes (I return to this in Section 11). Thus the case still seems Very Strong at this level.

I will call the highest level of claim “RNA First”. Here, I include the claim that RNA was the first kind of genetic biopolymer that stored specific sequences, and the claim that sequence replication preceded metabolism. This means there was no pre-RNA genetic polymer, that non-enzymatic replication of RNA oligomers could arise from prebiotic chemistry, and that no autocatalytic metabolic network of small molecules was required before sequence replication. The ubiquity of the ribosome and central dogma do not, by themselves, make a case for this higher level of claim, but I do mean to argue for the RNA First claim later in this paper.

## 3. Prebiotic Synthesis of RNA

It used to be claimed that RNA was too difficult to synthesize abiotically [18]. In 2004 Orgel [19] gave a gloomy view of the state of prebiotic chemistry of RNA (although he was generally supportive of the RNA World idea). But since then, there have been considerable advances. The likely route to synthesis of ribose involves the formose reaction, which converts formaldehyde to a complex mixture of sugars in which ribose is a fairly minor component [20]. However, it has been shown that in the presence of borate, the ribose fraction is increased relative to other pentoses and larger molecules [21,22,23]. Furthermore, sulphur dioxide, which is potentially present in the early Earth atmosphere due to release from volcanoes, also reacts with formaldehyde and glycolaldehyde and influences the formose reaction [24]. This chemistry is thought to occur on land in semi-arid conditions. This fits with other arguments suggesting that life evolved on land and that wet-dry cycling was important to drive condensation reactions [25]. However, recent work challenges the relevance of the formose reaction for ribose synthesis, and suggests that the major products may be branched ketoses, rather than linear aldoses like ribose [26]. So our understanding of the formose reaction is not as solid as we would like. Other pathways of ribose synthesis may be relevant, or nucleotides could be synthesized by pathways that do not require separate synthesis of ribose and nucleobases.

Prebiotic syntheses of nucleobases have been known for some time [19,27] although forming the glycosidic bond between ribose and the nucleobases appeared difficult. Alternative routes for synthesis of pyrimidine nucleotides have been found [28,29] that overcome this problem. This has become part of a more general cyanosulfidic chemistry that explains the synthesis of nucleotides alongside many other types of biologically relevant molecules including amino acids and lipids [30,31]. Other prebiotic mechanisms have also been found for synthesis of purine and pyrimidine nucleosides [32].

The reaction pathways proposed for prebiotic chemistry are significantly different from those used by today’s biochemistry, which opens up a heated debate. Some say we should use current biochemistry as a guide to the origin of life and look for prebiotic mechanisms for autotrophic carbon fixation following the pathways used in anaerobic bacteria [33,34]. Others say that synthesising life’s building blocks using the same pathways as extant biology is impossible without enzymes, and that prebiotic chemistry must have worked in a different way [35]. There is still no consensus on this.

Another important question is how nucleosides became phosphorylated. The most common form of phosphate is orthophosphate, PO_4_^3−^, which is difficult to react with organic molecules. It was argued some time ago that phosphates are an unlikely prebiotic reagent and that the first genetic polymers could not have contained phosphate ester backbones [36]. However, more recent evidence suggests that more reactive forms of phosphate were also available [37,38,39]. Nucleosides can also be phosphorylated by reaction with trimetaphosphate or diamidophosphate [40,41,42]. If phosphorylation occurs in the presence of borate, it is preferentially the 5′ carbon that is phosphorylated [43], hence borate also helps to understand why life makes use of 5′-activated nucleotides. Thus, it seems likely that phosphorylated nucleotides existed on the early Earth.

Polymerization of nucleotides is dependent on the state of phosphorylation of the 5′ and 3′ carbons of the nucleotides, and this affects the distribution of lengths of oligomers formed by random polymerization [44]. Studies have shown polymerization from 5′ monophosphates [45,46,47], 2′-3′ cyclic phosphates [48,49,50], and 5′ triphosphates [51]. Thus, the steps required for prebiotic synthesis of nucleotides and RNA oligomers are fairly well understood. Benner et al. [24] say that RNA can be formed via chemical steps that could “not *not* have happened” on the prebiotic Earth. Perhaps this is too confident, given the lack of consensus on the details of the organic chemistry [26]. We still have little idea of the concentrations of reagents that may have been reached on the prebiotic Earth, and we do not have strong constraints on the temperature and moisture content of the environment in which the relevant chemistry was occurring. I rate this part of the case as Strong, and I do not see a reason to doubt that a prebiotic supply of RNA was available. But I also acknowledge that I am not an organic chemist and my judgement in this area is only second-hand.

## 4. Non-Enzymatic Replication

Once the RNA World was established, it is assumed that sequence replication was catalyzed by polymerase ribozymes. However, it is likely that this was preceded by a stage of non-enzymatic replication, where RNA strands were synthesized using complementary strands as templates, without the presence of a polymerase ribozyme or a protein enzyme. If non-enzymatic replication occured, multiple copies of a sequence and its complement would arise in a mixure simultaneously. This is necessary if a polymerase ribozyme is to emerge from the mixture, because a single polymerase cannot copy itself—it needs other polymerases and complementary sequences to use as a templates.

The basic process of primer extension via addition of activated nucleotides has been clearly shown [8,9,52,53,54,55,56], although it remains rather slow and error prone. Most experiments assume there is activation of nucleotides by adding a leaving group to the 5′ carbon. This makes the extension of the sequence on the template thermodynamically favourable, and the templating process is driven in the forward direction. Most experiments use imidazole-activated nucleotides rather than the nucleotide triphosphates used in modern biology. The mechanism of primer extension involves the formation of a bridged 5′-5′ dimer, and the subsequent transfer of one half of the dimer onto the growing primer [55]. It has been shown that primer extension reactions can occur inside lipid vesicles, driven by a supply of activated single nucleotides from outside [56], which supports the idea that the first cells may have been heterotrophic, relying on abiotic synthesis of nucleotides in the environment.

It remains an open question whether primer extension occurs mostly one monomer at a time, or via addition of dimers and trimers, or via ligation of longer oligomers. If dimers and trimers are activated in situ then extension by ligation of dimers and trimers is possible [57,58] and may occur at a greater rate than monomer addition. Even if there is no direct addition of dimers and trimers, their presence may stabilize monomers in adjacent positions to the primer and may lead to a substantially increased rate of monomer addition [8].

All this makes the case for non-enzymatic replication Strong, but I do not yet rate it Very Strong because a number of significant issues remain. Firstly, it is not clear whether imidazole-activated nucleotides were present prebiotically. If replication did begin with imidazole-activated monomers it is then necessary to explain how (and why) life later changed to a triphosphate-activated system. It has been shown that ligase ribozymes working with imdazolide nucleotides can easily evolve to use triphosphate nucleotides [59], making it possible that this shift occurred after the origin of ribozymes. But it remains possible that future experiments will demonstrate non-enzymatic replication using triphosphates, in which case such a transition would not be required. Templated ligation may also be possible with 2′-3′ cyclic nucleotides [60], which would avoid the need for a prebiotic mechanism of creating 5′-activated nucleotides, but would still require a later transition to the use of triphosphates.

The large error rate in sequence copying is still a problem if there is to be a transition from non-enzymatic templating of random oligomers to ribozyme-catalyzed replication of specific sequences. It is known that the rate of extension after a previous mismatch pair is significantly slower than after a correct previous pair, which is known as stalling. This results in a net decrease of the average error rate in completed sequences, which may help to explain how the error threshold problem is avoided [54]. However, stalling also creates large numbers of incomplete sequences ending with errors, so this issue is not fully resolved. Another problem is that the rate of addition of C and G nucleotides is substantially higher than A and U, which causes a significant bias in sequence composition. The bias can be reduced by using 2-thiouridine (s2U) and inosine (I) instead of U and A [61], or by using conditions in which bridged dimers are sandwiched by downstream oligomers, or bridged oligimers allow extension by more than one base at a time [62].

**Figure 1:**
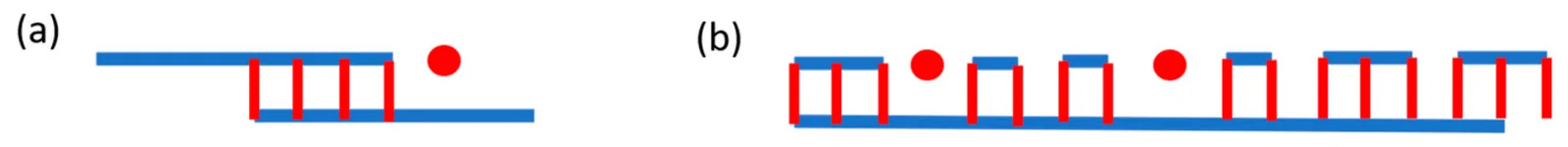
(**a**) Primer extension as envisaged in the virtual curcular genome mechanism. The red dot represents an activated nucleotide. The primer is extended one base at a time. (**b**) Replication of a full sequence by a linear replication mechanism involving ligation of many monomers, dimers and trimers bound to the same template. This would require in situ activation of all the fragments being ligated.

Another issue is that primer extension occurs at temperatures where double strands are strong and difficult to separate. It is therefore supposed that temperature cycling occurs between low temperatures where strands grow by templating and high temperatures where strands separate. Cycling of pH or ionic concentrations can also drive double-strand melting. The problem remains that reannealing of complementary strands formed in the previous cycle is much faster than growth of new complementary strands. If there are multiple copies of the same pair of complementary strands this causes inhibition of replication at a very low concentration [63]. The inhibition problem can be reduced if the reaction occurs in a viscous solvent [64], and in any case, inhibition is not such a problem if there is a mixture of partially overlapping fragments, rather than multiple copies of identical sequences. In this case, reannealing creates staggered double strands on which primer extension can continue, as in Figure 1a. It has been proposed that mixtures of overlapping fragments can form a virtual circular genome (VCG) [65]. In a VCG mixture, every strand would have a complementary strand with which it could pair and on which it could grow by primer extension. Thus, the whole sequence mixture could be sustainable. It was suggested that a sequence encoding a functional ribozyme could be encoded in such a sequence mixture.

An advantage of the VCG mechanism is that it can work even if the primer extension reaction is slow, because it only requires extension of one or two bases on each primer in one temperature cycle. Each primer can continue to grow in the next cycle after separation and reannealing to a new partner. However, in the VCG mechanism, each sequence is not copied from a single template; it is copied from several templates with which it is paired over mutiple growth cycles. This causes a problem that we call scrambling, which arises if the VCG base sequence contains repeated short sequences of a few bases. In this case, each fragment can pair with more than one different template, leading to the growth of sequences that are not contained in the original VCG sequence. Hence sequence information is lost [66]. The scrambling problem occurs even if the error rate in copying is zero, hence it is different from the error-threshold problem where sequence information is lost due to the accumulation of mismatch errors.

More recently, we have shown that if the mixture consists of sequences that form an irreducible cycle [67], then the sequence mixture continues to propagate accurately when the error rate is small, because only sequences that belong to the set can be synthesized from the existing templates. The simplest examples of patterns with two bases A and U are [A] + [U] (mutually complementary poly-A and poly-U strands) and [AU] (self-complementary AU repeats). Square brackets mean repeat the enclosed sequence multiple times and take all possible subsequences from this. Each pattern can be assigned a level $l_{0}$, which is the minimum length of word that is not repeated (where “word” refers to any short sequence of a few bases appearing within a longer sequence). For example, the pattern [AAU] + [AUU] is level 3, because the two-letter words AU and UA are repeated in the two halves of the pattern, but the 6 three-letter words are all unique (AAU, AUA, UAA, AUU, UUA, UAU). Table 2 lists all possible irreducible cycles up to level 4 with only two bases. Longer patterns with higher levels also exist. Simulations beginning from random oligomer sequences lose sequence diversity until they collapse to these irreducible cycles. Once an irreducible cycle is formed, the sequence pattern remains stable. Table 2 shows the number of runs out of 100 repeats that collapsed to each cycle, showing that shorter cycles with lower levels form more frequently. If there are four bases A, C, G and U, the number of possible cycles is very large and the cycles are somewhat longer. An example of a short cycle with four bases is the combination of [AC] repeats and [UG] repeats, which are mutually complementary. Four-base simulations starting with random oligomers were found to collapse to patterns with lengths between 1 and 32. We also tried simulations in which the mixture was initiated in a desired sequence pattern, and we asked whether the pattern remained stable without scrambling. Longer patterns tend to collapse to shorter ones. We found that patterns of length 12–20 that encode a single RNA hairpin loop can be stably replicated, but a pattern of length 44 encoding a hammerhead ribozyme can barely be replicated even with the most optimistic choice of rate parameters [67]. Encoding longer ribozymes than this, or encoding more than one ribozyme simultaneously on a VCG would not be possible.

Scrambling occurs because the VCG mechanism relies on reannealing of fragments produced in the previous cycle. A possible alternative is that long fragments with useful sequence information are rare in comparison to monomers, dimers and trimers. Hence, long sequences do not reanneal, and each long sequence can be covered by a mixture of the short fragments, as in Figure 1b. This possibility is suggested by experiments using in situ activated dimers [58]. In this case, a new long sequence is a copy of a single template sequence, so no scrambling occurs. We call this linear replication because the sequences are not required to form a closed virtual circle. In this case, mutliple independent functional sequences can be replicated simultaneously. However, this will only work if the ligation reaction is very efficient, so that a single long sequence is copied from a template, rather than several shorter sequences. Linear replication requires complete copying of the whole sequence in one temperature cycle, whereas the VCG mechanism requires extension by only one or two bases per cycle. Linear replication would also require the occasional extension of the product sequence beyond the end of the template (as indicated in Figure 1b) in order to counteract the inevitable tendency to shorten sequences. In fact, if the dominant mechanism uses bridged dimers, then the last base on the template cannot be copied. Linear replication would depend on ligation of in situ activated 3′-5′ dimers and trimers, which in some cases has been found to be slow in complarison to single-monomer extension via bridged dimers [62].

Current experimental work on non-enzyatic replication is making good progress, but there is still no clear demonstration of sustainable replication over multiple sequence generations, and no complete explanation of how sequence information could be accurately copied. These things must have been necessary if non-enzymatic replication were to give rise to self-replicating polymerase ribozymes, as the RNA World theory supposes.

## 5. Polymerase Ribozymes

There has been steady progress with laboratory studies of polymerase ribozymes and the performance of known sequences has been steadily improved by in vitro evolution. In 2001, Johnstone et al. [10] obtained a polymerase with the ability to extend a primer sequence by up to 14 bases, which was a record at that time. By 2013 a ribozyme had been developed capable of synthesis of a sequence over 200 bases long [11], which is longer than the ribozyme. However, copying was dependent on the template sequence, and the ribozyme could not replicate its own sequence. Since then, a ribozyme capable of exponential amplification of short RNAs by copying both complementary strands was obtained [68], and also a ribozyme capable of sustained replication of a hammerhead ribozyme sequence [69]. This demonstrates that the polymerase could operate with templates containing internal structure and that replication was sufficiently accurate to maintain the function of the hammerhead. Another significant improvement in sophistication is shown by the ribozyme in [70], which binds to the template at a specific promoter sequence and contains an additional domain that clamps around the template and increases processivity along the template.

Polymerases have also been developed that use trimer substrates instead of single monomers [71]. We saw above that dimers and trimers may be also important in non-enzymatic replication. Trimer substrates might help replication because they bind to templates with sufficient strength to disrupt the internal secondary structure of the template, which might otherwise inhibit the function of the polymerase. Binding of trimers to templates also prevents the reannealing of complementary template pairs, which matters because double strand formation by reannealing would also inhibit the polymerase. The recently-discovered QT45 polymerase [72] works with trimer substrates and is able to copy both itself and its complementary sequence, demonstrating the ability of ribozymes to self-replicate. The fact that this sequence is only 45 bases long is significant because shorter sequences are much more likely to arise in a random sequence mixture. The length of 45 is close to our estimate of the limit to the sequence length that could be replicated by the VCG mechanism [67]. Thus it begins to seem possible that a short polymerase can originate in a random sequence mixture with non-enzymatic replication, and that the gap from non-enzymatic to ribozyme-catalyzed replication can be bridged.

Of course, current polymerase ribozymes are not perfect. They still have limitations on the temperature and pH at which they function. They still are not as accurate as we might like, and still have relatively short life times in solution. In a prebiotic environment they would have to function in the presence of many other kinds of molecules without being inhibited. We might doubt whether current ribozymes would function in natural environments for any of these reasons. Nevertheless, the QT45 sequence is still recent, and it is likely that significant improvements to it will be made in future experiments. There are relatively few research groups working on ribozymes, so we are far from knowing the full range of possible polymerases in sequence space. There are likely to be many others that are better than those currently known. Given this, current results are already remarkably successful. Known polymerase ribozymes already do the jobs required of them by the RNA World theory. Thus, I rate this part of the case as Very Strong.

## 6. The Frequency of Functional Ribozymes in Sequence Space

I now turn to the claim of Meyer [17] that intelligent design is required to explain the OoL. His main point is that biological macromolecules contain information that cannot be created by chance. Although the Shannon information of a sequence can be defined mathematically, it is difficult to define information precisely in a way that is relevant to biological function. Nevertheless, at least qualitatively, we may say that “information” refers to the specification of a rare functional sequence relative to a large number of possible alternatives. Many RNAs and proteins have specific biological functions that are dependent on the details of their molecular sequence. How did these specific sequences with specific functions arise? Rather than asking “can information be generated by chance?”, I would prefer to phrase the question as “can prebiotic chemistry generate functional biological molecules with specified sequences?” I accept that a relatively small fraction of random nucleotide sequences will be a functional ribozyme and a relatively small fraction of random amino acid sequences will be a functional protein. So I agree with Meyer that the existence of functional ribozymes and proteins is something important that needs to be explained.

The argument against the chance production of functional sequences supposes that the probability of generating a functional sequence is very small—only 1 in $10^{B}$—where B is some big number. Then it is supposed that the number of attempts to make the desired molecule is $10^{A}$, where A is another big number. The number of times the desired molecule will occur is $10^{A-B}$. If A−B≫0, the desired molecule is almost guaranteed to be formed, but if A−B≪0, it will have a very small probability of being formed even once by chance. This logic makes sense, but the conclusion depends on what values we choose for A and B.

Meyer [17] (chapter 9) takes the example of a protein of length 150 with 20 types of amino acid, so the probability of a single sequence would be 1 in $20^{150}\;$= 1 in $10^{195}$. Rather than assuming only a single functional sequence, he cites work of Axe [73] which estimates the probability of forming a functional protein of length 150 as 1 in $10^{74}$, which is still extremely small. He then argues that amino acids can be linked either by a peptide bond or a non-peptide bond, which gives another $2^{149}\approx 10^{45}$ ways of making a polymer, only one of which has a backbone of regular peptide bonds. Finally, each amino acid is chiral, with either L or D forms, so this gives another $2^{150}\approx 10^{45}$ ways, only one of which is the desired homochiral protein. Thus the probability of making the functional protein is claimed to be 1 in $10^{74+45+45}=10^{164}$. He then estimates the number of possible attempts ([17] chap 10). He claims there are approximately $10^{80}$ elementary particles in the observable universe, the number of seconds since the big bang is $10^{17}$ and the maximum possible number of interactions per second is $10^{43}$ (this comes from consideration of the Planck time, a cosmological property of space-time). The maximum possible number of attempts is therefore $10^{80+17+43}=10^{140}$. His conclusion is that since $10^{140-164}\ll 1$, proteins could never arise by chance.

There are many problems with this argument. Meyer calls this estimate of A “extravangantly generous”. I would call it unreasonably large. It is irrelevant to bring in the size of the observable universe, the age of the universe, and the Planck time scale. Life evolved using only the available material on Earth in a time of only a few hundred million years after the Earth became habitable [74], and with realistic rates of chemical reactions for bond formation. My estimate of A would be much smaller, but Meyer claims that even with this unreasonably large A, it is still less than B, so life cannot evolve by chance.

I do not accept this conclusion for several reasons. Firstly, the earliest functional sequences were probably RNAs not proteins, so we should do the calculation with 4 nucleotides instead of 20 amino acids, which makes a big difference to B. Secondly, we should start with short ribozymes. I will use L=45 because this is the size of the QT45 RNA polymerase discussed in Section 5. The size of the sequence space is then $4^{45}=10^{27}$, a far cry from $10^{195}$ that Meyer began with. Nucleotides have L and D enantiomers and they can be linked with either 3′-5′ bonds or 2′-5′ bonds, so the problems of homochirality and bond choice exist for RNA in the same way as for proteins. However, these problems were already solved at the level of non-enzymatic replication of random oligomers prior to the origin of functional ribozymes (I discuss this in Section 9 below). Therefore I assume that there is a pool of random homochiral oligomers with the correct 3′-5′ linkages, so there is no additional combinatorial factor from these things. We still have a probability of 1 in $10^{27}$ for a ribozyme of length 45. If we used the extravagantly generous estimate of $10^{140}$ attempts, finding a ribozyme would be very easy. But we need to do better than this if we are to produce a convincing argument why chance formation of ribozymes is really possible.

**Figure 2:**
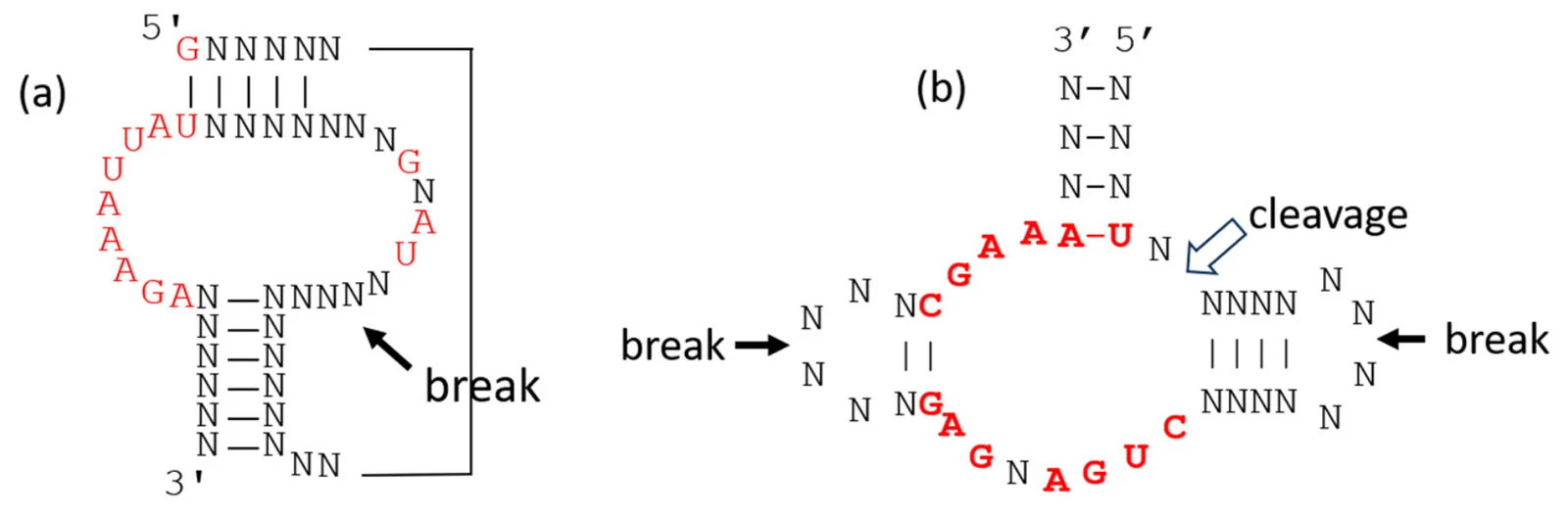
Conserved secondary structures of (**a**) a self-triphosphorylating ribozyme and (**b**) a hammerhead self-cleaving ribozyme used in calculations of ribozyme frequencies in random pools [75]. Red bases are conserved. N denotes variable bases. N-N pairs in stem regions must form base pairs. Additional bases may be inserted at break points without disrupting the structure.

Actually the frequency of functional ribozymes is much larger than 1 in $4^{L}$ because there are many different sequences that perform any given function. We know this because ribozymes of many kinds have been successfully isolated from random pools by in vitro selection. To develop a theoretical understanding of in vitro selection experiments we estimated the initial frequencies of several real ribozymes in random sequence pools [75]. The number of starting sequences in a typical experiment is only around $10^{15}$, but the frequency of functional ribozymes such as hammerheads and triphosphorylation ribozymes is around 1 in $10^{12}$ or $10^{13}$, so these sequences exist in the initial mixture and can be multiplied by the selection procedure. These numbers are calculated from knowledge of the number of matching base pairs in the conserved secondary structures and the number of single bases that must be specified for ribozyme function (see Figure 2).

The conserved structure puts constraints on the sequence, but there are many sequences that satisfy these constraints. In addition there is a large combinatorial factor W, which is the number of ways that random bases can be inserted at break points in the ribozyme structure without disrupting the function. W  may be thousands in some cases, which increases the frequency of functional sequences by several orders of magnitude, making them much easier to find in the experiment. Our estimates of functional sequence frequency are high enough to explain why these sequences are present in the initial random pool and how they can be recovered in the experiments [75], but to counter claims of intelligent design we do not need to worry about mathematical calculations because it is a fact that ribozymes of many kinds were successfully isolated in real experiments. This includes the QT45 polymerase and other longer polymerases, as well as the short ribozymes in Figure 2. This proves that the frequency of functional ribozymes is high enough that they exist by chance in a volume that is small enough to fit into the experimental apparatus (let alone the whole surface of the Earth or the whole observable universe). So A−B>0, which conclusively disproves the argument that functional sequences are too rare to form by chance. Hence I rate this aspect of the case as Very Strong. I also note that even those with a Christian viewpoint have not found Meyer’s argument for intelligent design convinving [76].

Something similar can also be said for protein sequences. Meyer considers a protein of length 150, but proteins much shorter that this could also have a function, and there could be fewer than 20 amino acids used in the first proteins [77], so the size of the sequence space for small proteins is not too large. Finding functional proteins is not impossible, but replication and evolution of proteins depends on the translation process, which I assume to have evolved at a later stage than RNA replication. The information in a protein sequence is specified by the RNA sequence, so Meyer’s question of how biological information originates must be answered with RNAs not proteins.

## 7. How Can Evolution Select for Ribozymes?

In vitro selection experiments prove that a small number of functional molecules is present in the initial random mixture, but this is only part of the story. Selection experiments use a procedure that separates functional from non-functional molecules and multiplies the functional ones before inputting them to the next round of selection. This procedure is intelligently designed by the experimenter (although the molecules themselves are not designed, because the experimenter does not know the structure of the functional molecules in advance). To counter the argument for intelligent design it is necessary to show that *natural* selection can cause the increase in frequency of the functional sequence without requiring *artificial* selection by an intelligent agent.

If evolution depended on replication by a polymerase that had itself evolved, then it could be argued that evolution by natural selection could not create the first polymerase, so prebiotic evolution would be a contradiction in terms. But this is not true if non-enzymatic RNA replication occured. As most random oligomers can be templates, oligomers copied non-enzymatically do not need to have functional information initially—i.e., they do not need to be a ribozyme or to do anything useful other than be a template. Selection on physical and chemical properties can act on RNA oligomers that do not have functional information. For example, an RNA oligomer has a free energy of association with its complementary strand, and hence a melting temperature for the double helix, which may be higher or lower than other sequences. Physical processes can select molecules with higher or lower strengths of double strand formation. Oligomers may also differ in their ability to act as templates, because of their different GC contents, or because of the possibility that internal structure could interfere with templating. Oligomers could also differ in their stability against degradation by UV light or in their strength of binding to mineral surfaces. If these kinds of selection on molecular properties are combined with replication, we have the possibility of evolution. Thus RNA oligomers can evolve, and a functional sequence can eventually appear by chance within a mixture of non-functional sequences.

The term “chemical evolution” is sometimes used to describe cases where selection acts on physical and chemical properties of sequences, but this term is problematic because it is used in different ways by different authors, and it is sometimes used in cases where no sequence replication is thought to be occurring. I will discuss possible meanings of chemical evolution in Section 10, and consider whether something akin to evolution could occur prior to sequence replication. But in this section, I am assuming that sequence replication is possible, and my use of “evolution” implies this.

Evolution with selection acting on physical and chemical properties of sequences is certainly possible, but I do not mean to imply that this is what drives the formation of functional sequences. It is better to say that functional sequences must arise by chance in spite of selection for physical and chemical properties. This point is related to what Meyer calls “predestination” ([17] chap 11). If there were interactions between monomers that favoured the formation of bonds between certain kinds of monomers, then polymerization chemistry would generate non-random sequences, as had been suggested in earlier work on proteins [78]. However, Meyer rules out predestination as a source of functional sequence information in nucleic acids because he says there are no bonds between the bases in the same strand. This is naïve because it does not account for stacking interactions between neighbouring bases, which depend on the order of bases in the sequence. As discussed above, there is a large GC bias in the sequences copied non-enzymatically. Work on non-enzymatic replication tries to avoid these sequence biases [61,62] because it is assumed that a sequence population with very high GC content would be less likely to contain functional ribozymes. The frequency of occurrence of possible secondary structures is dependent on base composition [79]. The likelihood of various kinds of ligation and recombination reactions also depends on base composition in complex ways [80]. The frequency of functional sequences can also be increased by selection for sequences that have a reduced tendency to aggregate [81]. Selection on physical and chemical properties can potentially influence the frequency of functional ribozymes within the mixture, but it is not clear whether the effect is positive or negative. Either way, many sequences will pass through this kind of selection, and this alone does not seem sufficient to guarantee the formation of a functional ribozyme. There is still much searching to be done to find functional sequences among the large number of sequences that pass the test of physical and chemical selection.

What we need to explain the origin of functional molecules is evolution, so we must think carefully how evolution works in the context of OoL. If a functional sequence such as a polymerase ribozyme arises in a random mixture, when can it cause its own selection? Since a polymerase cannot directly copy itself, it requires a mixture of sequences containing complementary minus strands and other copies of the polymerase plus strand. Other non-functional sequences in the mixture are parasites, because they take the time and resources of the polymerase without contributing anything. Parasites are inevitably produced by inaccurate copying of functional sequences, even if they are not initially present. Theory shows that in a well-mixed system, parasites increase in frequency until the polymerases are eliminated (except in the unachievable limit of absolutely zero error rate). There are two well-understood mechanisms by which this problem can be avoided [14,82,83,84,85]. Firstly in a system where polymerases act on neighbouring templates and where diffusion is slow (such as when strands are adsorbed on a 2d surface), polymerases survive up to a finite error threshold due to spatial clustering. Secondly, if polymerases are contained in compartments such as lipid vesicles, selection at the compartment level favours growth and division of compartments with more polymerases and fewer parasites, allowing survival of the polymerase up to a finite error threshold.

**Figure 3:**
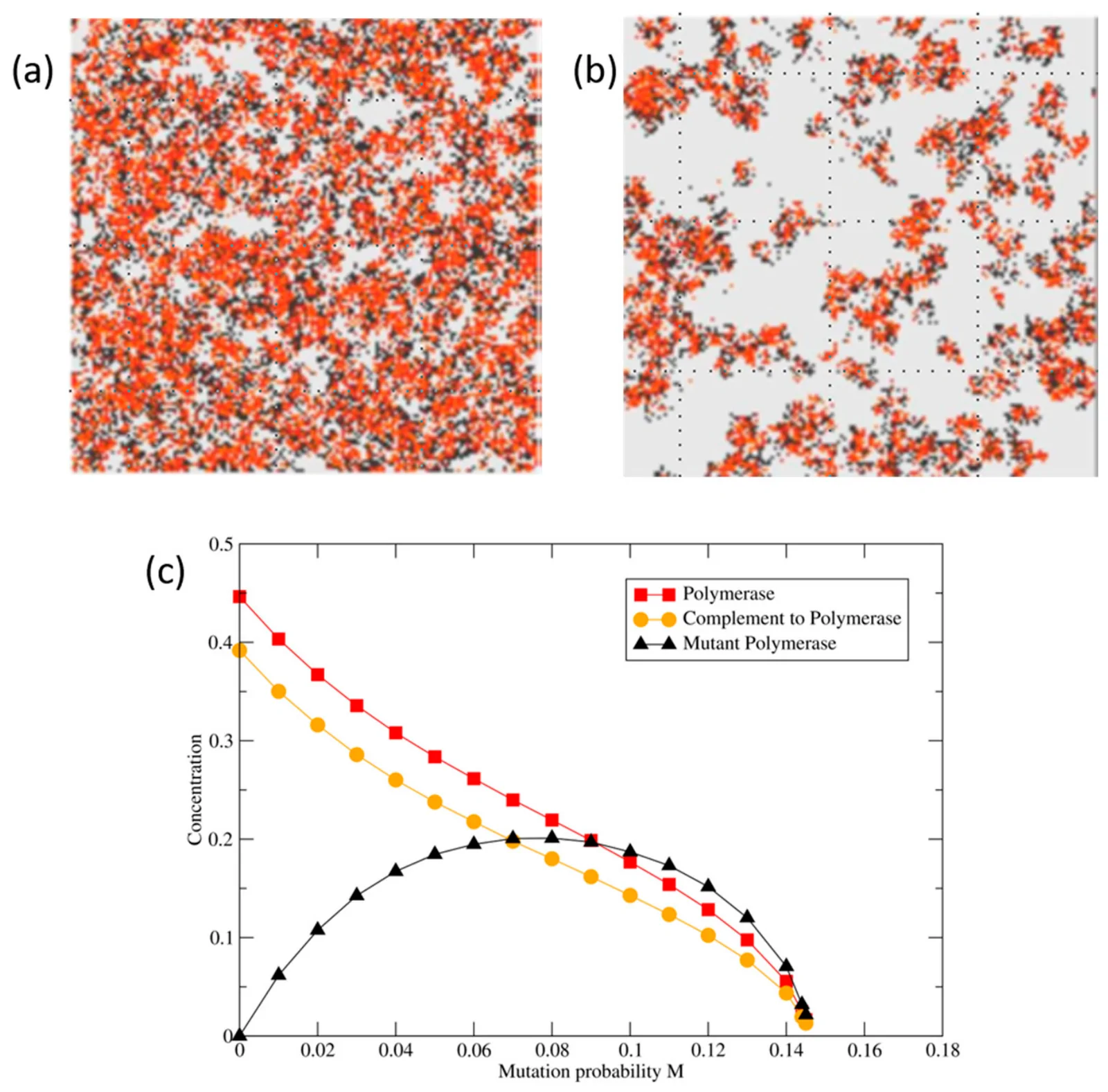
Model for replication of polymerases (red), complemenary sequences (orange) and parasites (black) on a spatial lattice (details in [83]) demonstrating survival of cooperative polymerases up to a finite error threshold. (**a**) M=0.08, (**b**) M=0.13, (**c**) Mean strand frequencies as a function of M.

A simple model illustrating spatial clustering of polymerases is shown in Figure 3. Each site on a square lattice may be occupied by one strand that is either a polymerase (P, red), a complement to a polymerase (C, orange) or a non-functional parasitic sequence (X, black), or it may be empty (grey). In each time step there is a probability that each P sequence copies the strand on one of its neighbouring sites. The new strand is placed in another vacant neighbouring site, if one exists (details in [83]). Copying a P or C produces an accurate copy (C or P) with probability 1−M and a mutant non-functional parasite X with probability M, where M is the probability of a deleterious mutation occurring somewhere in the whole sequence. Each strand also has a probability of destruction per time step, which creates new vacant sites. Clusters of sequences grow and shrink on the lattice due to birth and death events. This creates spatial patterns in which P and C sequences tend to be clustered (Figure 3a,b). Clustering favours cooperative polymerases, whereas it is disadvantageous to parasites because parasites require mixing with polymerases in order to be replicated. Figure 3c shows the average frequencies of P,C and X strands as a function of M, demonstrating that P and C sequences survive up to a finite error threshold around M=0.14 in this example (this value depends on the replication and death rates). In contrast, in a well-mixed model, where strands are repositioned on random sites every time step, P and C sequences do not survive at all unless *M* = 0.0.

We considered several extensions of this model. If there are both nucleotide synthetases and polymerase ribozymes, we showed that these can survive by joint clustering, even in cases where neither survives alone [83]. If parasites are produced that are much shorter than functional polymerases and therefore replicate faster, the polymerases still survive due to spatial clustering [84]. Comparison of the effects of vesicle compartments and spatial clustering showed that the minimumum replication rate required for polymerase survival was lower and the maximum tolerable error rate was higher in vesicles than with spatial clustering [85], suggesting that the scenario of RNA replication in vesicles is a likely one for early life. Models of vesicle compartments can naturally be extended to consider multiple types of functional ribozymes in the same vesicle, whereas spatial clustering of unlinked molecules becomes increasingly more difficult when the number of types of molecule increases, so vesicle compartments are important to allow evolution of more complex sets of mutually beneficial ribozymes.

A recent review discusses the importance of vesicles and other possible types of compartments [86] for early life. The competition of polymerases and parasites has been studied experimentally in a system of water-in-oil droplets in which polymerase proteins are translated from RNA genes by a cell-free translation system [87,88]. Hopefully it will be possible to improve experimental polymerase ribozymes to the point where a direct study of RNA-catalyzed RNA replication in compartments becomes possible without the need for a translation step.

Interesting questions remain as to how the first ribozymes were selected. It is not obvious that a polymerase must necessarily be the first ribozyme to arise. If non-enzymatic replication was sufficiently accurate to copy functional sequences, then ribozymes with other functions could precede polymerases. This would apply to a nucleotide synthetase that catalyzes any of the steps in the pathway of nucleotide synthesis [89], or a ribozyme that phosphorylates nucleotides [90,91], or forms imidazole-activated nucleotides. Ribozymes that modify lipids, leading to greater stability of vesicle membranes, are also good candidates for early ribozymes [92,93,94]. Ribozymes that inhibit mutations and increase the accuracy of replication have also been suggested [95].

All the ribozymes suggested in the previous paragraph have a function that would be generally beneficial to the sequence replication process, and which would help to support open-ended evolution of sequences with additional functions. In contrast, other kinds of ribozymes are known that function in a sequence-specific manner, including ligases [96] and recombinases [97] that can assemble new copies of their own sequence from fragments. These kinds of ribozymes show autocatalytic replication, but only if there is a continued supply of the fragments that are assembled. The fragments are not produced by the ribozyme, so these ribozymes would require replication of the fragments either by an efficient non-enzymatic process or by another polymerase ribozyme. These ribozymes would not support open-ended evolution of other functional sequences in the way that would be possible if there were a general polymerase ribozyme that worked on any template. Recent work also shows the possibility of very short sequences capable of templated ligation [98] and loop-closing ligation [99]. These appear simple enough that they might easily exist in a random mixture. They would not support open-ended evolution, but it is possible that such short ribozymes might have helped facilitate the origin of a longer general polymerase.

For most cases of ribozyme evolution it is important to have selection at the level of compartments/protocells. Therefore it is important to understand how a ribozyme in a protocell affects the properties of the protocell. Simple models such as [85] assume that a protocell divides in two when the number of strands inside reaches a specified maximum. If this is true, then rapid sequence replication automatically causes rapid cell division, so a polymerase proliferates because protocells containing polymerases multiply faster. To go beyond this, we need a better understanding of the mechanism of cell division. If cell division is triggered by external events, such as changes in temperature, drying of the pond, or addition of extra lipids, rather than internally triggered by growth of the cell, then cell division is less strongly correlated with the properties of sequences in the cell. We have begun to study a model for growth and division of vesicles containing an internal autocatalytic reaction [100]. Cells grow if additional lipids can be added to the membrane from the environment and if the autocatalytic reaction maintains an osmotic pressure that causes the cell to swell. Cell division depends on the relative rate of growth of volume and membrane area, and on the build up of asymmetry in lipids between outer and inner leaflets of the bilayer, which causes membrane curvature and favours cell division.

To summarize this section, both theory and experiment show that natural selection for ribozymes in compartments is possible, assuming that sequences can be copied non-enzymatically initially, and later by ribozymes after polymerase ribozymes arise. This defeats claims that prebiotic evolution is impossible and that intelligent design is necessary. Therefore, I rate this part of the case as Very Strong. The possible functions of the first ribozymes and the way in which molecular functions influence cell properties remain interesting research questions.

## 8. RNA and the Origin of Metabolism

Although the RNA-first view of OoL focuses on sequence replication and tends to ignore metabolism, it is necessary to bring metabolism into the picture if we are to understand the origin of modern cells. One link between RNA and metabolism that was noted long ago is that many of the cofactors used by protein enzymes contain nucleic acid moieties. This includes ATP, Coenzyme A, NAD, FAD and SAM [101,102,103,104,105]. This suggests that the cofactors arose in an RNA World with ribozyme catalysts and that they continued to be used by protein enzymes after the origin of protein synthesis. There have also been some studies demonstrating ribozyme catalysis of some of the steps of synthesis of these cofactors [106,107].

It has been argued [102] that once a critical mass of catalysts makes use of a cofactor then it is very difficult to change the cofactor because it would simultaneously affect the reactions performed by many catalysts. Hence the cofactors are preserved unchanged even though the catalysts have changed from ribozymes to proteins. Cofactors are strong evidence for the existence of an RNA World with multiple ribozymes catalyzing multiple metabolic reactions.

These arguments may be true, but they feel unsatisfying, because they try to justify why ribozymes with diverse functions are no longer present in current organisms, without proving that these ribozymes actually existed originally. It would be more convincing if ribozymes could be selected in vitro that catalyze a wide range of metabolic reactions currently catalyzed by enzymes. But there are few examples of this. Most known ribozymes catalyze reactions related to RNA ligation, polymerization, cleavage and splicing, rather than the reactions of small-molecule substrates that are essential in modern metabolism. This may simply indicate that few studies have attempted to isolate such metabolic ribozymes. But it also raises the possibility that the diversity of metabolic ribozymes in the RNA World was never very large. The RNA-first argument assumes the first organisms were heterotrophic, and relied on chemically synthesized nucleotides and on the inherent ability of oligomers to carry out templating reactions. In this case, the ribozymes that would be immediately useful are those that promote RNA synthesis and replication—exactly those kinds of ribozymes that have already been found by in vitro selection. If the RNA world had a limited metabolic scope and relied on a bountiful supply of reagents synthesized by prebiotic chemistry, it would be confined to environments where prebiotic chemistry was particularly favourable to it. If such an RNA World managed to invent protein synthesis, it may be that protein-catalyzed reactions enabled it to become more independent of the environment and eventually to become fully autotrophic. In this case, the pathways that are central to modern metabolism would have arisen after the origin of protein synthesis, and may not have existed in the RNA World.

Researchers advocating a metabolism-first rather than an RNA-first view approach metabolism in different ways. One approach is to try to show that the reactions in current metabolic pathways can occur without catalysis by proteins or ribozymes. There has been some success in this when reactions are considered separately [108,109,110] but little success in showing that a complete autocatalytic cycle can occur with only small molecules. There is a minimal sense in which metabolic reactions must be possible without enzymes, since an enzyme can only be evolved for a reaction that is chemically possible in the first place. But finding a slow rate of individual reaction steps is not the same as saying that all the steps can work together to produce an autocatalytic cycle that can sustain a protocell in absence of enzymes.

One specific question related to the origin of metabolism is why ATP is used extensively as a means of transfer of phosphate groups to other molecules, and why the adenine base of ATP is necessary at all if the primary function of the molecule is to transfer a phosphate. The simplest explanation of this is that the primary function of ATP was *not* for the transfer of phosphates in metabolism, but for the synthesis of RNA. The A base is then esential to allow base pairing in double strands, and the triphosphate may have been the activating group for RNA polymerization and templating reactions. If ATP were already present, it could then have been recruited to metabolic roles when metabolism evolved at a later stage. It has been found that ATP is more suited chemically for phosphate transfer than other nucleotide triphosphates [111], which explains why ATP is used more extensively in metabolism than other NTPs, but this does not explain why a nucleotide of any kind should be used for this purpose rather than a simpler molecule, or why ATP should fortuitously have the ability to form base pairs, unless RNA base pairing were already essential for RNA replication that was occurring prior to the origin of metabolism.

Other arguments for metabolism-first scenarios are based on the idea of autocatalytic sets. These are sets of molecules and reactions that can sustain themselves because all reactions are catalyzed by some molecule that is present in the set. Theoretical models show that autocatalytic sets are likely to emerge in random reaction networks if there is high diversity of molecules and the frequency of catalyzed reactions is sufficient [112,113,114]. However, self-sustaining autocatalytic cycles of small molecules have not been found experimentally.

An idea similar to autocatalytic sets is the GARD model, which considers lipid micelles composed of mixtures of many kinds of amphiphilic molecules [115,116] that are able to promote entry of further molecules from solution into the micelles. Computer simulations of this model show that certain mixtures are able to grow in a way that conserves the composition of the mixture. But compositional inheritance of lipids has not been convincingly demonstrated in experiment. The existence of protocells containing replicating nucleic acid sequences seems to be a necessary step to understand the origin of modern cells, but it is not necessary that the protocell membrane should have a heritable lipid composition. It could simply make use of whatever lipids were frequent in the environment without requiring precise control of the composition. Furthermore, even if inheritance of a specific lipid composition were demonstrated in an experiment with micelles or lipid vesicles, it is not clear how this would make the origin of replicating nucleic acids any easier. Proposing a stage of compositional inheritance prior to sequence replication seems to add an unproven and unnecessary additional step to the RNA-first viewpoint.

**Figure 4:**
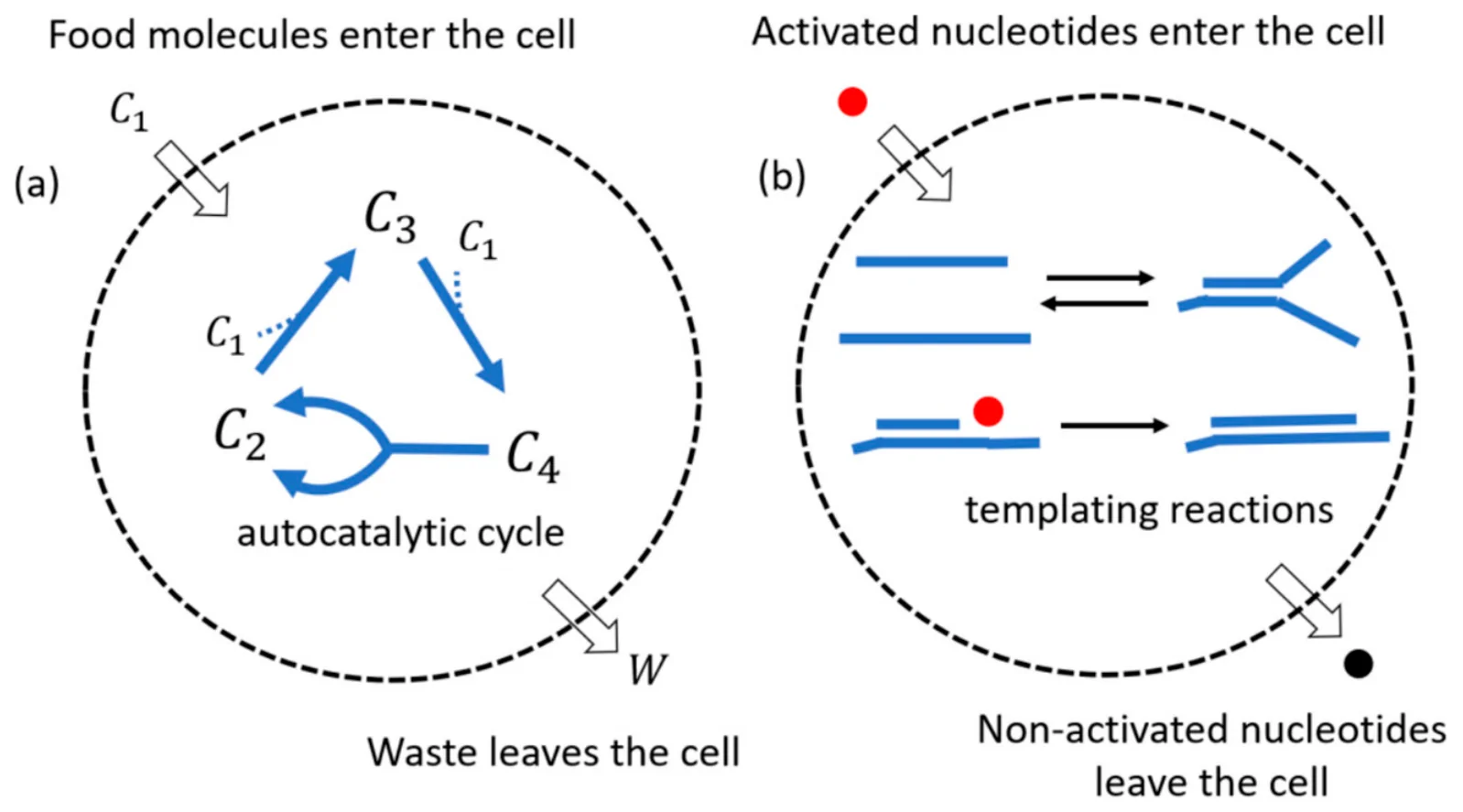
(**a**) A protocell driven by an autocatalytic cycle of small molecules that forms a metabolism. The bifurcating arrow indicates that two copies of $C_{2}$ are formed by splitting a $C_{4}$ molecule. The cycle as a whole is autocatalytic—the concentrations of $C_{2},\;C_{3}$ and $C_{4}$ all increase if there is a continued supply of $C_{1}$. (**b**) A protocell in which templating of RNA oligomers occurs. The figure shows formation and melting of double strands and primer extension by addition of an activated nucleotide. The RNA process and the small-molecule cycle are conceptually similar. The cycle of nucleotide activation, templating and breakdown of oligomers is itself a metabolim [119].

Recently, I tried to set aside the idea of RNA sequence replication (at least temporarily) and to take seriously the possibility that there might exist protocells containing an autocatalytic reaction system without any kind of genetic molecule. Figure 4a shows a protocell driven by a supply of a food molecule $C_{1}$ that enters through the membrane. Catalysts $C_{2},C_{3},C_{4}$ inside the protocell form an autocatalytic cycle that converts $C_{1}$ into more catalysts. The catalysts also react to form waste molecules W that leave the cell. The reaction cycle shown in Figure 4a is actually the Breslow cycle of the formose reaction [117], where $C_{1}$ is formaldehyde, and $C_{2}$ is glycolaldehyde. We asked what properties the autocatalytic reaction must have if it is to drive the sustainable growth and division of a protocell? A key requirement is that the autocatalytic reaction must sustain itself in a non-equilibrium active state inside the cell, but the same reaction must not occur in the environment outside the cell [118,119]. We have called this condition inside-outside stability (IO Stability). A requirement for IO stability is that the rate of the autocatalytic cycle is proportional to the square of the catalyst concentration (second-order) rather than the catalyst concentration (first-order). Cells with an IO stable reaction system can continue to grow and divide while the food source in the environment remains in a non-reactive state [100,119]. But if the reaction system is not IO stable, the autocatalytic process either dies out, or it starts spontaneously both inside and outside the cell, so the required difference between the cell and environment is not maintained.

We have given several theoretical examples of reaction networks that are IO stable [119], but we do not know of real chemical systems that fit these reaction schemes. The formose reaction is one of the only known prebiotically-relevant autocatalytic cycles, but this is first-order and not IO stable, so we claim that it cannot be used as the internal reaction that drives protocell growth, even though it may be important for synthesis of sugars outside the cell, as discussed in Section 3, and even though temporary growth of droplets driven by the formose reaction has been seen experimentally [120].

An unexpected conclusion of our work on IO stability was that we were led back to RNA replication, despite having intended to set it aside for a while. We showed that a system of non-enzymatic replication of RNA oligomers in a protocell driven by a supply of activated nucleotides from outside is IO stable because the templating rate depends on the square of the strand concentration [119]. Thus the RNA replication system has the required properties to drive protocell growth and division, as shown in Figure 4b. We should think of nucleotide activation and templating of oligomers as *being* a metabolism in themselves, rather than thinking of sequence replication as a separate thing that must be driven by some other process that we call metabolism. The lack of experimental examples of small-molecule autocatalytic reaction systems is a major weakness of theories that propose metabolism first without sequence replication. But the fact that oligomer replication is an autocatalytic process that has the properties required for a metabolism, means that the RNA-first theory unexpectedly relates to the origin of metabolism as well as sequence replication. For this reason, and because of the long-recognized importance of nucleotide-related cofactors, I rate this aspect of the case for the RNA World as Strong.

## 9. RNA and the Origin of Homochirality

Nucleotides, amino acids and many other biological molecules have two enantiomers of opposite chirality, and nature has chosen to make use of one of them almost exclusively. The origin of homochirality of biological macromolecules is a fundamental part of the OoL problem. The problem can be divided into physical and chemical questions. Physical questions concern whether the supply of molecules available for the origin of life initially had a chiral bias due to an abiotic physical mechanism. Chemical questions concern how autocatalytic reaction mechanisms amplify chiral bias and lead to homochiral systems.

The most relevant physical source of bias would be the action of circularly polarized light in space, which can bias molecular synthesis in protoplanetary disks and can explain the observation of chiral biases of the amino acids found in meteorites [121,122]. However, the molecular supply from meteorites may be neglible in comparison to chemical synthesis occurring on Earth. Tiny amounts of nucleobases have been found in meteorites but essentially no nucleotides, so synthesis of nucleotides by Earth-based chemistry seems to be necessary for OoL (see Section 3). This means the origin of homochirality is essentially a chemistry question. It has long been understood that autocatalytic chemical reaction systems can be unstable to spontaneous chiral symmetry breaking [123,124,125]. If molecules of each enantiomer help to catalyze synthesis of further molecules of the same enantiomer and molecules of opposite enantiomers inhibit one another in some way, and if the system is out of equilibrium, with continued synthesis of new molecules and breakdown or loss of old, then it is possible for a 50:50 mixture of L and D enantiomers to be unstable and to spontaneously become dominated by one or other enantiomer. When the reaction mechanism is unstable to symmetry breaking, small excesses of either L or D are amplified exponentially, so tiny biases created by stochastic fluctuations are sufficient to initiate symmetry breaking (whether or not there are also small initial biases due to physical reasons).

The essential part of the problem is to understand the chemical mechanism that causes exponential amplification of the enantiomeric excess, and to understand which types of molecules are involved. Experimentally, symmetry breaking is seen in the Soai reaction [126], and possible mechanisms related to amino acid dimerization have been shown recently [127], but here I will argue that nucleic acids became chiral before amino acids or other biomolecules, and that the mechanism responsible is templating of RNA oligomers, rather than synthesis of individual nucleotides. It is known that homochiral RNA strands are good templates whereas strands of mixed chirality are not, and that nucleotides of the opposite chirality tend to inhibit templating reactions [128,129]. Several computational models of RNA replication have shown that spontaneous symmetry breaking occurs [130,131,132]. Strands become homochiral, even though the supply of single monomers is racemic. Recent work [132] identifies three ways by which a 50:50 mixture of strands can become unstable—either by racemization of single monomers, by inhibition between strands of opposite chirality, or by non-linear competition between opposite chirality strands. The third mechanism occurs when replication takes place in conditions where the total strand concentration is fixed, as is expected in a vesicle that is growing and dividing, or in any situation where there is a shared resource that limits the total concentration of L plus D sequences.

For an environment with separate local compartments, such as vesicles or spatially distributed pores in rocks, we expect symmetry breaking to occur randomly in each one, so that wherever we look we shall see homochiral strands locally, but there will still be a mixture of L and D strands on a global scale. This situation can arise at the level of oligomers having no function. Since mixed LD strands are eliminated by templating before the origin of functional ribozyme sequences, we do not need to add an extra combinatorial factor for chirality when calculating the probability of forming a functional sequence (as discussed in Section 6). Once functional ribozymes evolve, they will do so in strands that are either all-L or all-D. As ribozyme functions will depend on the chance origin of rare sequences, they will not be equivalent in L and D vesicles, meaning that one form will eventually outcompete the other on a global scale. Once ribozymes evolve that control nucleotide synthesis, single nucleotides will be synthesized of the enantiomer that is required by the ribozymes, so homochirality will be strongly reinforced by the origin of functional sequences, although it arises initially before function evolves.

In this scenario, RNA becomes chiral before amino acids and peptides, and chirality is transferred to proteins because the tRNAs and rRNAs that control protein synthesis are already chiral, and the translation mechanism works with amino acids of one enantiomer. Several other arguments also suggest that nucleic acids were the first kind of biomolecule to become chiral [133]. I rate our understaning of the origin of homochirality in terms of the RNA-first view as Strong, but it will be important to find experimental demonstrations that the symmetry breaking mechanisms expected from simulations do actually occur. This will require templating experiments that are sustainable over long times.

We see that non-enzymatic templating plays many important roles in OoL. Not only does it allow for the origin of genetics and evolution (Section 7), it is also linked strongly to the origin of metabolism (Section 8) and homochirality (this section).

## 10. Chemical Evolution—Why Did Nature Choose RNA?

In the discussion of evolution in Section 7, I was assuming that RNA strands existed and could replicate, and hence that evolution of specific RNA sequences could occur. But this leaves several lower-level questions that might be linked to evolution. Is the RNA molecule itself a product of evolution? Was there some other kind of polymer that preceded RNA? Was there some process that we might call chemical evolution that was required before RNA could form? Why did nature chose to use RNA in the early stages of life, rather than any of the other nucleic-acid-like polymers that might have performed a similar role?

The ubiquity of RNAs in the translation process tells us that RNA was chosen as a foundation for sequence evolution very early in the history of life. It is clear that RNA can act as both a gene and a catalyst, but it is unlikely to be the only kind of molecule that could do this, so it is not obvious why nature chose RNA in particular. There are many alternative genetic polymers with different backbones. These are known as xeno nucleic acids (XNAs). Examples include threose nucleic acid (TNA), peptide nucleic acid (PNA), hexitol nucleic acid (HNA), acyclic threoninol nucleic acid (aTNA) and many others [134,135,136]. Some XNAs can form hybrid double strands with RNA and DNA, which raises the possibility of transfer of sequence information between polymers. Some XNAs can perform nonenzymatic templated ligation and primer extension [137]. Catalytic sequences of some types of XNA have been found [138]. In addition to differences in polymer backbone, there are many other possible base pairs that could have been used on a backbone of RNA or another polymer [139,140,141], raising the question of why A, C, G and U(T) were chosen by nature.

If we ever find alien life on another planet, I would bet that it will also be based on replication of some kind of polymer with a small set of possible monomers, but I would not bet that it will be based on RNA in particular, because there seem to be so many other polymers that could work in a similar way. Nevertheless, on Earth, RNA was selected, and it seems unlikely that this choice was entirely random. In other words, it is tempting to suppose that RNA was “better” for some reason than many of the other possible polymers that existed alongside it originally. What made it better?

Could it simply be that ribonucleotides were the most frequent type of monomer in the prebiotic mixture? Ribonucleotides must have been reasonably common, or there would be no hope of starting an RNA World. But there must have been many other kinds of monomers capable of polymerization, and we have no reason to expect that ribonucleotides outnumbered these alternatives. Could it be that RNA oligomers were more stable than the alternatives? This seems unconvincing because cleavage of the RNA backbone occurs relatively easily. Could it be that RNA sequences are better catalysts than sequences of other kinds of polymers? We have an incomplete understanding of the range of catalytic functions that can be carried out by XNA sequences, but there seems no reason to suppose that this range is more limited than it is for RNA. Also, the choice of genetic polymer could not be made using magical foresight of the range of catalytic functions that would be possible to evolve if this polymer were chosen. The polymer replication system has to be in place before sequences made from this polymer can start to evolve.

For this reason, I think that RNA was selected because it was efficient at performing non-enzymatic templating with short oligomers before there were any functional sequences. What made it efficient at templating? The rates of templated ligation and primer extension must have been relatively quick and relatively accurate compared to other polymers. But the evidence for this seems weak, given that current experiments with nonenzymatic RNA replication are still slow and inaccurate. Maybe RNA structures were more stable than those of other polymers? The free energies of double strand formation must have been relatively strong to allow templating. In fact, base pair stacking in RNA is quite strong, with 2 or 3 kT per base pair, so that even rather short double strands are stable for long times. But it is not clear that this stacking is stronger than it is for other kinds of polymer. Furthermore, double strands need to melt to allow continued replication, so base pair stacking cannot be too strong. Base pairing within a single strand must be strong enough to allow stable secondary and tertiary structures, but not too strong to prevent molecular unfolding and refolding. Another possibility is that there was some efficient kind of activation chemistry that worked with ribonucleotides rather than other kinds of monomers. We don’t know. All of these arguments seem uncertain, so I rate our current understanding of why nature chose RNA as Weak.

It is sometimes argued that there was another kind of replicating polymer prior to RNA which was later replaced by RNA [142,143]. The possibility of hybrid strands between different polymers means that sequence information could in principle be transferred between polymers. We know that this can occur in both directions between RNA and DNA, so could it be possible that this occurred between a pre-RNA polymer and RNA? I find this unlikely because transfer of sequence is not the same as transfer of function. It is possible to store the sequence of ribosomal RNA on a DNA genome, but this does not mean that a DNA strand with the same base sequence as rRNA will function as a ribosome. Functional details depend on the tertiary structure of the molecule, which will not be the same for different polymers, even if the base sequence is the same. For this reason, once strands with catalytic function have evolved, life cannot change its genetic polymer without restarting evolution from scratch. Therefore, I argue that RNA was the first kind of molecule in which functional catalytic sequences evolved. This argument does not prevent the subsequent evolution of DNA as a genetic storage molecule or the evolution of proteins as an additional form of catalyst, since in neither of these cases is an RNA function transferred to another kind of molecule.

So I believe there was no pre-RNA genetic polymer, but there may have been other kinds of XNA oligomers alongside RNA oligomers before there was any genetics. I envisage a competition between non-enzymatic replication of different kinds of oligomers before there were functional sequences. Strands with mixed monomers of different kinds are likely to be poor templates relative to uniform strands of a single kind of monomer. A process of selection from heterogeneous monomers to homogeneous oligomers will occur [144,145]. Computational models show that the best kind of template sequences can win this competition and reach high frequency relative to alternative XNAs [130]. The mechanism is similar to the origin of homochirality, where one or other of the homochiral strands wins the competition. But in the case of RNA versus XNA, the polymers are different, so the competition is not equal and there is no exact symmetry to begin with. I presume that RNA won this competition at the stage of non-functional oligomers, but we are still far from understanding exactly why RNA did win.

We might be tempted to call this kind of selection and competition between different kinds of polymers “chemical evolution”. But this is potentially misleading without additional explanation. I have argued [146] that if this term is to be meaningful, it has to be distinguished from “just chemistry” with no evolution, and from “biological evolution”, which applies to genes and proteins in modern organisms. I suggested that chemical evolution should be used when selection acts on physical and chemical properties which all sequences possess, whereas biological evolution should apply to discussion of biological functions that are possessed by only a small fraction of sequences (i.e., information is required to specifiy them, in Meyer’s sense). Additionally chemical evolution should be applied when discussing sequences that are short enough to be synthesized by random polymerization as well as by templating, whereas biological evolution should apply when discussing long sequences that can only be re-synthesized by copying a previous template. If we consider competition between RNA and other XNA polymers, we could say that oligomers of each kind of polymer could show chemical evolution relative to other oligomers of the same kind, but this does not mean that one kind of polymer evolved from another. Therefore I am reluctant to describle the selection of RNA relative to other XNAs as evolution.

Historically, chemical evolution has been used in a context where there is no evolution at all, e.g., Lemmon [147] defines it as “the study of the biologically relevant chemistry that preceded Darwinian evolution”. I would prefer to call this simply prebiotic chemistry. In recent years, chemical evolution has been used to refer to experiments that look at at the changes of concentrations of products in mixtures of chemicals with condensation reactions [148,149]. These experiments show interesting chemical effects, but they do not include replication or any other means of heredity, so I would prefer to call them “just chemistry” rather than evolution.

In the context of prebiotic synthesis of RNA, chemical evolution has been used to label the argument that “RNA does not arise directly from simple building blocks in a prebiotic milieu but from iterative cycles of diversity generation, selective retention, and chemical transformation within heterogeneous organic mixtures” [150]. This was contrasted with a direct synthesis model, which “views prebiotic chemistry as a sequence of well-defined, productive chemical reactions that transform simple building blocks into RNA via serial and parallel pathways”. To me, both these quotes are descriptions of prebiotic chemistry, not evolution. The so-called chemical evolution model emphasizes iterative cycles and selective retention, and implies that properties are inherited from one cycle to the next in some way, even without sequence replication. Such things may be possible, but it has not yet been shown to what extent heredity could occur in absence of sequence replication. The chemical evolution model views RNA as being too complex to form initially without some prior iterative process, but it has not been shown how such an iterative process would make it easier to synthesize RNA than by a direct synthesis model. There are also claims that RNA has properties that show that it must have evolved, such as recalcitrance [151] (the increased stability to hydrolysis of sequences that fold to a compact tertiary structure) and “goldilocks” behaviour [152] (where folded RNA structures have increased stability in a certain range of Mg^2+^ concentration). These properties may be useful properties for biological molecules, and they may be part of the reason why RNA was selected by nature. But these are are inherent physical and chemical properties of RNA (and probably of other water-soluble polymers too). To say that these properties evolved seems a misuse of the word evolution. Current RNA properties are not inherited from some earlier RNA that lacked these properties, or from some other kind of polymer that passed the properties on to RNA.

Another different way to define chemical evolution is to note that biological evolution, as usually conceived, requires genetics and individuals, but it is possible to define chemical evolution as evolution in cases which lack these [153]. For example, replicating RNAs that were not confined in clearly separated compartments would lack individuals, and heredity by some means other than sequence replication would lack genetics (e.g., composomes in the GARD model [115,116]). It has also been suggested that evolution can function by selection acting on molecular stability, with possible help from autocatalysis, without the necessity of sequence replication [154]. An example where one sequence has a large 100-fold advantage in stability was considered in [154]. But it is not clear why one sequence should have such a huge advantage, or why this stable sequence should have a useful function, even if it were very stable. On the other hand, if sequence replication occurs, differences in fitness are amplified over many generations. Hence small fitness differences yield large differences in sequence frequencies (as shown in the mathematical models of [146]). In other words, evolution with sequence replication is much more effective than without it.

One reason that I am somewhat picky about the use of “chemical evolution” is because I think evolution is essential to explain the origin of functional biological molecules, and because creationists and intelligent design advocates have repeatedly tried to cast doubt on evolution. Therefore, it is important to be clear about what evolution is, and it does not help for scientists to use the word evolution when discussing other kinds of chemistry where there is no real evolution. Meyer [17] (chap 2) also uses the term chemical evolution and says that it is insufficient to explain OoL, but he uses it in reference to early theories of Oparin about the steps from chemistry to the first cells, and not in the modern contexts mentioned above. What Meyer sees as impossible to explain scientifically is the origin of biological function. This is the distinction between biological and chemical evolution in my language [146]. Replication, heredity, and natural selection can occur without functional information, so it is not true that prebiotic evolution is a contradiction in terms. Within such a replicating mixture, a functional molecule, that *does* have sequence information, can arise. It is then important that the functional molecule should have some effect that leads to its own selection, and its increase in frequency. I discussed ways in which this can occur in Section 7. In my view, this requires evolution with sequence replication, and we cannot explain it with only prebiotic chemistry or with versions of chemical evolution that do not include replication.

## 11. How Did the RNA-Protein Relationship Arise?

We have not yet brought proteins into the story, but it is clear that proteins became essential to life relatively early. In the RNA World and RNA-first views, ribozymes were functional before proteins, and ribosomes evolved in the RNA World as a means of protein synthesis. But some would say that it is wrong to consider RNA as preceding proteins, because the two kinds of polymers coevolved and proteins were always essential for RNA function from the beginning. In this context it is important to distinguish peptides formed by random amino acid polymerization (or by some mechanism other than translation of RNA) from proteins having a specific sequence specified by translation of RNA. I will call an amino acid sequence a protein only in the latter case, otherwise it is a peptide. It is virtually impossible to argue that proteins were present from the beginning, whereas it is more reasonable to suppose peptides were always present.

The only way to claim that encoded proteins coevolved with RNAs from the beginning is to claim that the ribosome itself was the first kind of ribozyme and that polymerase ribozymes never existed, which has actually been suggested [155,156,157]. In this view, the first polymerases were proteins made by the ribosome. If ribosomal RNA arose without a polymerase ribozyme, then non-enzymatic replication must have been efficient and accurate enough to copy long ribosomal RNA sequences. This seems unlikely. Even the central core of the ribosome is much longer than the simpler ribozymes considered above. Presumably the original core of the ribosome catalyzed peptide synthesis by transfer of amino acids between proto-tRNAs, even if there was initially no codon-anticodon interaction with an mRNA. Thus, in addition to ribosomal RNA, the origin of translation requires the existence of tRNA-like molecules and some means of charging these with amino acids. This is one of the most difficult steps in early evolution, even if we suppose a polymerase ribozyme did exist first, and it would appear almost impossible if we had to leap from nothing to ribosomal protein synthesis without passing through a stage with ribozyme-catalyzed RNA replication.

The RNA World view supposes that polymerase ribozymes existed at an early stage and were later replaced by protein polymerases after the invention of translation. The lack of polymerase ribozymes in nature is frustrating if one is trying to reconstruct the steps that occurred in early evolution, but it is unavoidable in this standard view. A tantalizing suggestion is that polymerase ribozymes did not disappear completely, and that ribosomes are the remnants of early polymerase ribozymes that acquired a new role in protein synthesis. A recent variant of this is the idea of a “riboreplisome” that was a single molecule with linked domains that carried out RNA replication and protein synthesis [158]. These things appear unlikely to me, so I will stick with the assumption that there were polymerase ribozymes (and probably other ribozymes too) prior to the origin of translation. It is then necessary to understand why there was selection for evolution of a ribosome within an RNA World.

It has been suggested that the peptidyl transferase centre in the core of the large ribosomal subunit is the most ancient part of the ribosome, which predates the mRNA-decoding and translation functions located in the small ribosomal subunit [159,160,161]. If so, the ribsomal core was selected initially as a means to synthesize random (non-encoded) peptides, in which case we need to understand what were the benefits to RNA of synthesizing random peptides. There is some evidence that peptides with cationic and hydrophobic amino acids, like KKVVVVVV, can interact in a beneficial way with polymerase ribozymes [162], probably because aggregates form between positively charged peptides and negatively charged RNAs. It was also argued that ribosomal protein sequences can do this [163], but it seems likely that the effect depends only on the average amino acid properties rather than the specific amino acid sequence. Polyester and depsipetide polymers with cationic amino acid side groups have also been observed to stabilize folded RNA structures [164]. Cationic peptides also influenced the function of hammerhead ribozymes in coacervate droplets [165], although the overall catalytic rate of the ribozymes in the droplets was slower than when in free solution.

By preforming in vitro selection experiments in the presence of specific peptides, it is possible to isolate ribozymes for triphosphorylation that make use of these peptides [166,167]. However, unmodified peptides did not benefit ribozyme activity more than 3-fold [167], and only one peptide that by serendipidy contained a polyaromatic hydrocarbon showed much stronger benefits to RNA function, more than 100-fold [166]. Therefore, these experiments do not suggest that peptides are important for a functional RNA to evolve, or that the catalytic ability of ribozymes that interact with peptides is necessarily better than those that do not. Also, these experiments use multiple copies of the same peptide, whereas we would expect mixtures of random peptide sequences prior to the origin of translation. More experiments of this type will be needed in order to ask whether random peptides can significantly improve the function of ribozymes under typical conditions and whether additional functions of ribozyme-peptide complexes can be found that go beyond what ribozymes can do alone. If so, the argument for selection of the core of the ribosome would begin to seem more concrete.

Many possible early functions of peptides alongside RNA have been recently reviewed [77,168], emphasizing the possibility of self assembly of short peptides into amyloid-like structures, and suggesting that these structures may bias condensation reactions to generate peptides of like sequence. If possible, this would allow some degree of amino acid sequence evolution before translation from an RNA sequence was possible.

It could be the case that early cationic peptides also improved the function of the first ribosomes, but if random peptides already exsited, and RNA replication was controlled by polymerase ribozymes, it is not possible to select for additional ribosome-catalyzed peptide synthesis if the only benefit of the peptides is to the ribosome itself—why then would we need a ribosome at all? The peptides must have benefited a polymerase ribozyme, or another pre-exsiting ribozyme with a function other than peptide synthesis. I already referred to several suggestions that early peptides formed coacervates with RNA, and that this was beneficial because of mutual stabilization of the two kinds of polymer, or because the peptides facilitated ribozyme function. An interesting further possibility is that interaction of early peptides with RNA oligomers facilitated non-enzymatic RNA replication. If this were the case, the mutualistic interaction of the two polymers could indeed have begun very early. But as far as I am aware, nobody has investigated this. There are suggestions that RNA-peptide coacervates could function as reproducing entities [169] in a similar way to the catalytic micelles envisaged in the GARD model [170]. If such coacervate droplets existed, it would be possible that sequences catalyzing a proto-ribosomal mechanism of peptide synthesis could be selected within the droplets, i.e., protein synthesis would directly benefit the proto-ribozomes themselves rather than a separate ribozyme. However, this would only work if there were efficient non-enzymatic replication of the RNAs. Thus, a possible beneficial role of peptides in non-enzymatic RNA replication would be an interesting avenue to pursue from this point of view.

The origin of a system to translate an mRNA sequence is much more complicated than simply selecting for random peptide synthesis. One key step is the attachment of amino acids to the end of an RNA, as occurs with tRNAs in the modern translation process. Ribozymes able to self-aminoacylate [171] and to aminoacylate tRNAs [172,173] have been obtained. There are also suggestions that early tRNAs could have been minihelices with single hairpin loops [174,175]. Additionally, linking an amino acid or a short peptide to an RNA might have improved the range of ribozyme functions beyond that of pure RNA, so it might be possible to select for aminoacylation of RNAs because these molecules had a direct benefit rather than only being necessary as an intermediate step in protein synthesis. But all these arguments seem very speculative.

There is a large literature on evolution of the genetic code itself, looking at changes in the code after an initial translation system already came into being [176,177,178,179,180,181]. This work addresses questions related to which amino acids should be used by biology, which order they were added to the code, and why certain codons were assigned to certain amino acids. The question of the nature of the LUCA, the last universal common ancestor of all current cells, is often linked to the evolution of the genetic code. It has been argued that early cells underwent frequent horizontal gene transfer and that the sharing of genes was extremely important. An organism can only make use of a gene transferred from another organism if it uses the same genetic code. So this may be an important reason why all domains of life ended up sharing the canonical code. These things go beyond the scope of the present review.

Once a system allowing translation from RNA to protein sequences evolved, it is easy to see that there were many possible encoded proteins with beneficial functions and that there must have been great advantages in possessing genes for these functions. But the primary question for OoL is how and why the protein-RNA relationship got started in the first place. How far could RNA-based life have come if protein synthesis had not been invented? Was it inevitable that ribosomal protein synthesis would evolve? What were the functions of random peptides and the first encoded proteins? Our understanding of these things still seems only Weak.

## 12. Conclusions

The RNA World is the most complete and well-supported theory for the Origin of Life that we have, although it is still far from complete. The prebiotic chemistry by which RNA can be synthesized is increasingly well understood. There is solid experimental evidence that RNA can support non-enzymatic replication and that polymerase ribozymes can replicate functional sequences, including themselves. In vitro selection experiments demonstrate that functional ribozymes are relatively frequent in sequence space. Evolutionary theory demonstrates how cooperative polymerase ribozymes can be selected. Experiments demonstrate replication inside vesicles, hinting at how the first protocells could have functioned. The use of nucleotides as cofactors in current metabolism tells us that current metabolism arose within an RNA World. A system of RNA templating in a protocell driven by an external supply of activated nucleotides is a non-equilibrium reaction system that satisfies the requirements for a metabolism. A system of oligomers with templated replication undergoes spontaneous symmetry breaking to give homochiral strands. All these aspects of the OoL are successfully explained in a coherent way by viewing RNA as the central player in the OoL story. This is not just a vague story, it is backed up by experiments showing that RNA can really do the things required of it by the theory.

In Section 2, I distinguished the RNA World claim, that RNA functioned as a gene and a catalyst and that polymerase ribozymes catalyzed RNA replcation, from the RNA-first claim, that RNA was the first form of genetic polymer and that RNA sequence replication arose without the prior existence of a small-molecule metabolism or self-replicating micelles or droplets. I feel that current evidence supports the RNA-first claim in addition to the RNA World claim. Section 10 pointed to the difficulty of transfering the function of a molecular catalyst from one kind of backbone to another, making it seem highly unlikely that genetics got started first with some pre-RNA polymer. The claim that replication preceded metabolism is supported by the many experiments showing both non-enzymatic and ribozyme-catalyzed replication (Section 4 and Section 5) and the scarcity of experimental evidence for the alternatives. Evidence that would potentially change this conclusion might come in several forms. Metabolism-first scenarios would be greatly strengthened if it were demonstrated that complete autocatalytic cycles could be sustained using only small molecules without requiring biomolecular catalysts with specific sequences. Scenarios involving micelles or coacervate droplets first would be greatly strengthened by experimental demonstration of self-sustained growth and division in such systems, and by a clearer understanding of how sequence replication could eventually arise within the droplets. Scenarios proposing the importance of chemical evolution would be greatly strengthened by demonstration of how heredity could occur without sequence replication, and explanation of how a chemical evolution process would facilitate the eventual formation of replicating nucleic acids if it is argued that these cannot form directly from prebiotic chemistry. But in absence of any of these, I will continue to advocate RNA World and RNA-first. The RNA-first view is certainly not a fallacy, as has sometimes been claimed [182].

As scientists in the OoL field, we need to retain a degree of caution and humility in our claims because we can never go back to see exactly what happened. But we should not be over-cautious. We should not let scientific disagreements within the field give the impression that scientists are still clueless about OoL. As the OoL is a key part of our scientific understanding of how the world works and how we came to be here, it is of considerable interest to the general public. Scientists should take some responsibility to explain the science clearly to the public, stating what is known and admitting where the limits of our understanding lie. But those limits do not leave room for wild fantasies and miracles. Mainstream science views life as something that can be explained from the laws of physics and chemistry without the need for mysterious vital forces, intelligent design, or supernatural effects. The OoL community accepts this, but tends to take it for granted. We should remind ourselves why we we take this physicalist view of the world and what is the evidence that supports it, and we should be willing to defend the scientific case. Otherwise science becomes ignored in this world full of fake news and religious conservatism.

## Figures and Tables

**Table 1:** Steps in the case for the RNA World.

Topic	Strength of the Case
The ribosome and the central dogma	Very Strong
Prebiotic synthesis of RNA	Strong
Is non-enzymatic replication possible?	Strong
Are polymerase ribozymes possible?	Very Strong
Do ribozyme sequences exist with sufficient frequency?	Very Strong
Can evolution select for ribozyme function?	Very Strong
The role of RNA in the origin of metabolism The role of RNA in the origin of homochirality Why did Nature choose RNA? How did the Protein-RNA relationship arise?	Strong Strong Weak Weak

**Table 2:** Possible Irreducible Sets up to level 4 with sequences of A and U bases, showing the number of runs that formed this pattern starting from random sequences (out of 100 repeat runs). These data are the same as Table 1 in [67], but this table was wrongly formatted in the earlier publication.

Level $l_{0}$	Number of Words	Cycle Length	Generators	Number of Runs
1	2	1	[A] + [U]	20
1	2	2	[AU]	17
2	4	4	[AAUU]	5
3	6	3	[AAU] + [AUU]	11
3	6	6	[AAAUUU] [AAUAUU] [AAUUAU]	10 9 6
3	8	4	[AAAU] + [AUUU]	5
3	8	8	[AAAUAUUU] [AAAUUUAU]	4 8
4	8	8	[AAAAUUUU] [AAUAUUAU]	1 0
4	10	5	[AAAAU] + [AUUUU]	2
4	10	10	[AAAAUAUUUU] [AAAAUUUUAU] [AAAUAUUUAU]	0 2 0
4	12	12	[AAAAUAUUUUAU] [AAAUAAUUAUUU] [AAAUUUAUUAAU]	0 0 0
4	14	14	[AAAAUAAUUAUUUU] [AAAAUUUUAUUAAU]	0 0

## Data Availability

There are no new data with this article.
